# Zoonotic Transmission of Vaccine-Derived *Bordetella bronchiseptica*

**DOI:** 10.1093/ofid/ofad421

**Published:** 2023-08-07

**Authors:** Imke Kraai, Marjolein Knoester, Erik Bathoorn, Martijn Bakker, Marcel Nijland

**Affiliations:** Department of Hematology, University of Groningen, University Medical Center Groningen, Groningen, the Netherlands; Department of Medical Microbiology and Infection Prevention, University of Groningen, University Medical Center Groningen, Groningen, the Netherlands; Department of Medical Microbiology and Infection Prevention, University of Groningen, University Medical Center Groningen, Groningen, the Netherlands; Department of Hematology, University of Groningen, University Medical Center Groningen, Groningen, the Netherlands; Department of Hematology, University of Groningen, University Medical Center Groningen, Groningen, the Netherlands

**Keywords:** *Bordetella bronchiseptica*, live attenuated vaccination, zoonotic transmission

## Abstract

We describe a unique case of a 43-year-old-female with a Bordetella bronchiseptica infection caused by zoonotic transmission following vaccination of her dog. With this report, we want to raise awareness of potential zoonotic transmission of live attenuated vaccines from animals to patients with impaired immunity.

*Bordetella bronchiseptica* is an aerobic gram-negative bacterium. In cats and dogs, *B. bronchiseptica* is highly contagious, leading to bronchitis (kennel cough). In humans, *B. bronchiseptica* infections are rare. Patients with impaired immunity are at increased risk of colonization and opportunistic infections with *B. bronchiseptica* [1, 2]. Live attenuated vaccines for *B. bronchiseptica* are available for intranasal administration to cats and dogs. Although considered safe, these live attenuated vaccines, under the right conditions, might cause zoonotic infections. Confirmed cases of vaccine-derived zoonotic *B. bronchiseptica* infections are rare [3].

Herein, we describe a 43-year-old Caucasian female with a history of axial spondylarthritis who developed a *B. bronchiseptica* infection following vaccination of her dog with a live attenuated vaccine. The patient received the tumor necrosis factor alpha (TNF-α) inhibitor etanercept at a weekly dose of 50 mg for the past 5 years. Two weeks after intranasal vaccination of her dog with a live attenuated *B. bronchiseptica* vaccine (Nobivac KC, MSD Animal Health, USA), the patient started developing bronchitis with malaise and mild fever, which did not abide during the following week (Figure 1). At presentation to our clinic, she had a normal blood count and a normal C-reactive protein level. A blood culture and chest x-ray were normal. A multiplex polymerase chain reaction showed no viral infections but indicated a *Bordetella* species infection. The patient received azithromycin tablets 500 mg once daily for 3 days. A sputum culture showed the growth of *B. bronchiseptica* on charcoal agar. The patient suggested the potential zoonotic transmission of *B. bronchiseptica* following vaccination of her dog. Comparison of core genome multilocus sequence typing, based on short-read sequencing as described previously [4], using an ad hoc scheme including 4121 target genes (Supplementary Tables 1 and 2) showed only 3 allelic differences between the sputum sample isolate UMCGBbp2 and the *B. bronchiseptica* vaccine (batch A135E01) strain UMCGBbv1, thus confirming the vaccine as the source of the infection. The patient was treated with trimethoprim/sulfamethoxazole 960 mg bid for 14 days, after which her symptoms gradually improved.

**Figure 1.**
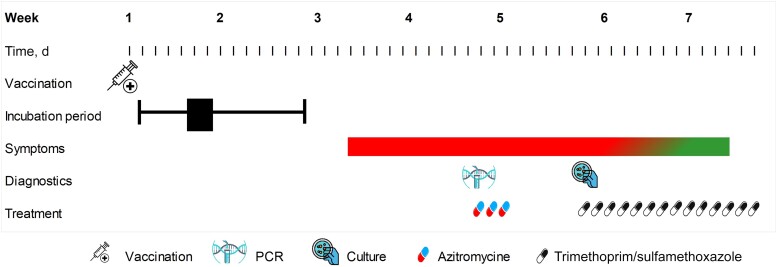
Timeline of *B. bronchiseptica* vaccine administration to a dog until first symptoms in a patient receiving a tumor necrosis factor–α inhibitor. Abbreviation: PCR, polymerase chain reaction.

To our knowledge, this is the first report of a confirmed zoonotic transmission (dog to human) of a live attenuated *B. bronchiseptica* vaccine. Although a primo infection of the patient at the time of vaccination of her dog cannot be fully ruled out, this is considered less likely as the median incubation time for *B. bronchiseptica* infections in animals is 5–6 days and our patient developed symptoms >14 days after vaccination [5]. The vaccine contains the live bacterium *B. bronchiseptica* strain B-C2. The vaccine differs from other *B. bronchiseptica* strains because certain molecules are missing, making it less virulent. Occasional side effects in animals include sneezing and coughing. Because shedding of bacteria may last for up to a year after vaccination, the European Medicines Agency advises against vaccinating animals of patients with impaired immunity. At the time of infection, the patient was receiving a TNF-α inhibitor. TNF-α inhibitors increase the risk of serious infections up to 2-fold, and reports suggest that there is an increased risk of opportunistic infections [6].

It is unclear whether our patient is unique or if zoonotic transmissions remain widely unrecognized or unreported. With this report, we want to raise awareness of the potential zoonotic transmission of live attenuated vaccines from animals to patients with impaired immunity.

## Supplementary Material

ofad421_Supplementary_Data

## Supplementary Data

Supplementary materials are available at *Open Forum Infectious Diseases* online. Consisting of data provided by the authors to benefit the reader, the posted materials are not copyedited and are the sole responsibility of the authors, so questions or comments should be addressed to the corresponding author.
